# A comprehensive systematic review of pharmacological and non-pharmacological depression interventions for patients on dialysis

**DOI:** 10.1177/20503121251353028

**Published:** 2025-08-23

**Authors:** Ahyeon Cho, Tammy Tran, Laura Telfer, Ahmad Matarneh, Sundus Sardar, Nasrollah Ghahramani

**Affiliations:** 1 Pennsylvania State University College of Medicine, Hershey, PA, USA; 2 Division of Nephrology, Department of Medicine, Penn State Health Milton S. Hershey Medical Center, Hershey, PA, USA; 3 Department of Medicine, Penn State Health Milton S. Hershey Medical Center, Hershey, PA, USA

**Keywords:** dialysis, major depressive disorder, depression intervention

## Abstract

**Background:**

Depression affects 38%–80% of end-stage renal disease patients on dialysis, causing increased hospitalizations, treatment nonadherence, and mortality rates. While various interventions have been researched, a comprehensive assessment remains necessary to address the psychological burden effectively.

**Objective:**

To assess previous research from 2017 to 2023 on the efficacy of pharmacological and non-pharmacological interventions for depressive symptoms in this group.

**Methods:**

A systematic review was performed across PubMed, ScienceDirect, Clinical Key, and Web of Science. Citations for inclusion and abstract extraction were assessed and confirmed by two independent researchers. Inclusion criteria consisted of clinical trials, randomized controlled trials, and prospective studies written in English. We excluded studies that were review articles, case reports, or editorials, or did not examine antidepressants, exercise, or other mental health interventions in dialysis patients. To assess risk of bias, the Risk of Bias 2 and the Risk of Bias in Non-randomized Studies of Interventions tools were utilized. Depressive symptoms were measured using different scales.

**Results:**

Among 911 screened citations, 30 articles were included, involving 1815 participants across 17 countries. Publications on antidepressant medication (*n* = 4), exercise (*n* = 9), music therapy (*n* = 4), and psychotherapy (*n* = 13) were included.

**Conclusion:**

While interventions like antidepressants, intradialytic exercise, music therapy, and psychotherapy show potential for managing depression in dialysis patients, small sample sizes, lack of control groups, and short treatment durations continue to limit current studies. Future research should focus on multicenter trials with larger, more diversified populations and stronger study designs.

## Background

End-stage renal disease (ESRD) presents a complex challenge, with studies reporting that 38%–80% of dialysis patients experience major depressive disorder (MDD).^1^ Disrupted eating patterns, combined with the restrictive dietary requirements of ESRD management, contribute to a declining nutritional status and physical health.^2^ Similarly, post-dialysis fatigue leads to increased dependency and a reduction in autonomy, impeding individuals from engaging in activities that foster a sense of purpose and fulfillment.^3^ The psychological burden of managing ESRD is further worsened by the financial strain associated with treatment costs.^4^ Due to loss of employment from physical restraints, decreased earnings, and high medical expenses, patients on dialysis continue to struggle financially with insufficient reimbursements.^5^ The perpetual cycle of stress and diminished motivation triggers a cascade of physiological responses, including sustained elevation of body mass index and C-reactive protein levels, which exacerbate mood disturbances and compromise overall resilience.^6^ In essence, the complex interplay between psychological distress and the demands of the ESRD treatment creates a vicious cycle, where diminished motivation and accumulating stressors combine to undermine patients’ health outcomes. The aftermath of mood disturbances extends across multiple dimensions, increasing hospitalization rates, hindering medication adherence, reducing attendance at medical appointments, and ultimately leading to increased mortality rates.^2,7,8^

Mitigating this cycle requires a multifaceted approach that prioritizes both medical and psychological support, emphasizing the importance of comprehensive care models that address the holistic needs of ESRD patients. According to the American College of Physicians’ Guideline for Nonpharmacologic and Pharmacologic Treatments of Adults in the Acute Phase of MDD, antidepressant medication and psychotherapy remain the standard treatments for MDD.^9^ Due to limited high-quality evidence, combination therapy with selective serotonin reuptake inhibitors (SSRIs) and cognitive behavioral therapy (CBT) is loosely suggested for dialysis patients, emphasizing the need for individualized treatment approaches.^10–13^ This study reviews recent literature to ensure that it reflects the most current research and clinical practices as treatment and patient care continue to evolve in this area. Given the prevalence of nonadherence to antidepressants among patients with chronic illness, this study aims to compare both psychosocial and pharmacological treatments, including antidepressants, exercise, psychotherapy, and music therapy.^14^ Furthermore, this analysis explores the specific benefits of each intervention, recognizing that individualized treatment is increasingly favored for this patient population.

## Method

### Search criteria

The Preferred Reporting Items for Systematic Reviews and Meta-Analysis (PRISMA) extension for systematic review was the guideline used for this study.^15,16^ Literature from PubMed, Science Direct, Clinical Key, and Web of Science were attained, starting from January 2017 to December 2023. A combination of Medical Subject Headings and keyword searches was utilized, applying a language filter to include only English-language articles (“MDD,” “depression,” or “depressive disorder”), (“Dialysis” or “hemodialysis”) AND (“depression treatment” or “depression interventions”) were phrases used to search related articles. See Supplemental Appendix 1 for further details on search strategies for each database.

### Inclusion and exclusion criteria

*The including criteria*:

Clinical trials, randomized controlled trials (RCTs), and prospective studies.

Written in English.

*The exclusion criteria*:

Guidelines, review articles, systematic review or meta-analysis, case report, book chapter, editorial, opinion, or commentary.

Non-English

Does not include antidepressant, exercise, or other intervention demonstrating effects on mental health.

Does not include dialysis patients

### Selection process

The following studies were extracted into a database and were organized by two reviewers (A.C. and T.T.) to decrease selection bias. Two authors examined full-text articles of potentially relevant articles to assess their eligibility. All disagreements were settled by a third reviewer (L.T.) and were documented.

### Outcomes

The primary outcome measured was the change in depression scale scores as determined by each study’s chosen assessment tool, which reflects the depressive symptoms before and after treatment.

### Data extraction

Two authors (A.C. and T.T.) completed the data extraction, utilizing standardized data extraction method from Microsoft Word. Extracted data included the first author and publication year, country of origin, study design, sample size and group allocation, type of intervention, depression scale used, primary outcomes with reported statistical values, and key findings and limitations. All final data were summarized in Table 1 (overview of study characteristics, intervention type, methodology, and key results) and Supplemental Appendix 2.

**Table 1.** Overview of depression interventions in ESRD patients with major depressive disorder.

First author (reference)	Year	Country	Depression scale	Interventions	Control (*n*)	Intervention (*n*)	*p* value	Conclusions	Limitations
Antidepressants									
Mehrotra et al.^19^	2019	USA	QIDS-C	Multicenter, parallel-group, open-label, RCT (Intradialytic CBT treatment) versus (Sertraline) Ten sessions over 12 weeks. Sertraline dosage started at 25 mg/day and titrated to 200 mg/day if tolerated	CBT group; 60 out of 120	Sertraline group; 60 out of 120	Sertraline group had decreased QIDS-C scores – 1.84 (95% CI, −3.54 to −0.13); *p* = 0.035 between groups	Significant reduction for depression scores in sertraline group compared to CBT group. Although there were more adverse events associated with the sertraline group	No long-term evaluation. Not compared to no-intervention control group
Friedli et al.^20^	2017	UK	BDI-II, MADRS	Randomized double-blind placebo-controlled trial (Sertraline) versus (Placebo) over 6 months. Began with an initial daily dose of 50 mg. Follow up at 2 weeks and at 2, 4, and 6 months. The psychiatrist could adjust the dosage to a maximum of 100 mg at the 2 and 4 month evaluations	13 out 15	8 out of 15	Difference of 1.89; 95% CI, −2.7 to 6.5; *p* = 0.20 between groups	Significant reduction in depressive scores in both intervention and control groups compared to baseline. No significance between sertraline and placebo groups	Small sample size, high dropout rate, recruitment was difficult
Kauffman et al.^21^	2022	USA	PHQ-9	Prospective interventional study, 20 mg of fluoxetine was prescribed qdaily for 2 weeks and then increased to 90 mg qweekly for 10 weeks. For those with inadequate response, dose was adjusted to 180 mg	None	16	Pre–post *p* = 0.002	For patients on chronic hemodialysis, once a week fluoxetine, when directly observed, appears to be a tolerable and successful treatment. Significant decrease in depression	Limited sample size, modestly elevated baseline PHQ-9 scores, no comparison group, and short treatment duration. Population predominantly female and African Americans
Guirguis et al.^22^	2020	UK	BDI-II, PHQ-9	Prospective multicenter, longitudinal cohort study. Prescribed antidepressants were reviewed. Citalopram was most common (16 patients, 39%) at 10–40 mg, followed by fluoxetine (nine patients, 22%) at 20–40 mg, and sertraline (six patients, 15%) at 50 mg. Mirtazapine and venlafaxine were each used by two patients (5%); escitalopram by two patients (5%). Less commonly prescribed were paroxetine, dothiepin, nortriptyline, and duloxetine (one patient each, 2%). Follow-up ranged from 6 to 15 months	None	41	Pre–post: BDI-II: *p* = 0.015 PHQ-9: *p* = 0.091	Antidepressant management for hemodialysis patients was suboptimal, characterized by ineffective choice of medication and its dosage, and insufficient follow-up, indicating non-compliance with NICE guidelines. Mixed results with significant reduction in BDI-II score but no significance with PHQ-9 score	Short length of study, population composed of elderly, and functionally impaired
Exercise									
Nakamura-Taira et al.^23^	2021	Japan	PHQ-9	Quasi-cluster randomized, open-label controlled trial (resistance exercise group) versus (stretching group (control)) for 6 months. Depressive symptoms and cognitive function were assessed at months 3, 6, and 12	21 out 42	21 out 42	*p* > 0.05 between two groups	No significant differences were found in depression scores	Gender ratio in each cluster differed, aware of their group assignment, two facilities
Zhou et al.^24^	2020	Qatar	CESDS	RCT (exergame group (virtually supervised exercise program)) or (in-person nursing supervised exercise group (control)) over 4 weeks	36 out 73	37 out 73	*p* = 0.246 between groups	Both groups experienced significant reductions in depression scores, with no difference between them. The exergame group reported positive experiences, emphasizing fun, safety, and helpful sensor feedback	Relatively low sample size
Turoń-Skrzypińska et al.^25^	2023	Poland	BDI-II	RCT for 20 min (VR exercises) versus (no intervention) during dialysis sessions over 3 months	46 out 85	39 out 85	Between group *p* = 0.202	Significant reduction of depression scores in the experimental group compared to baseline. Significant increase in depression scores in control group compared to baseline	Small sample size, only one dialysis center due to COVID pandemic
Ortega-Pérez de Villar et al.^26^	2020	Spain	CES-D	RCT (intradialytic group (control)) versus (home-based group) completed a 16 week combined exercise program three times per week	22 out 46	24 out 46	*p* = 0.017 pre–post treatment for intervention group *p* = 0.081 between groups	Significant reduction in depression in both groups compared to baseline. No significant differences between intervention and control group	High number of dropouts
Sheshadri et al.^27^	2020	USA	CESDS	Pilot RCT (walking therapy) versus (standard care). Pedometers were utilized with step goals per weekly or continued with 3 months	30 out 60	30 out 60	*p* = 0.1 between groups	No significant difference in depressive symptom burden or severity on the DSI and vitality scale	Limited due to study location, walking is not the only physical activity
Kauric Klein^28^	2022	USA	PHQ-9	Nonrandomized controlled trial (yoga group) versus (standard care). Participated in online sessions three times a week, with outcomes assessed after 4 weeks	12 out 31	19 out 31	*p* = 0.086 (intervention versus control)	No significant reduction in depression in intervention group compared to standard care. Significant reduction in depression in intervention group compared to their baseline	Small sample size, duration of study reduced from 12 to 4 weeks due to difficult recruitment, high dropout rates, lack of randomization
Grigoriou et al.^29^	2021	Greece	ZSDS, BDI	Prospective interventional study. This exercise program was supervised for 9 months. Aerobic cycling and resistance training during hemodialysis were incorporated	None	20	Pre–post: ZSDS: *p* < 0.01 BDI: *p* = 0.077	Significant reduction in ZSDS depression scores compared to baseline. No significance based on BDI scores	Patients were their own control, moderate sample size
Parent-Roberge et al.^30^	2021	Canada	BDI	Prospective interventional study; combination of aerobic and resistance training during dialysis were performed for 6 months	None	25	Pre–post *p* = 0.006	Significant reduction in depressive symptoms	One-arm design, small sample size, high dropout rate
Zhou et al.^31^	2023	China	HADS-D	Prospective interventional study; completed a 12 week intradialytic exercise program, performing 30 min sessions three times weekly during each dialysis session for the first 2 h	None	75	Pre–post *p* < 0.001	Significant reduction in depressive symptoms over time	Single-center study, no control group, lack of randomization, outcome not measured at 6 month follow-up due to COVID-19
Psychotherapy									
Picariello et al.^32^	2021	England	PHQ-9	RCT (CBT for fatigue (BReF) intervention) versus (waiting list group (control))	12 out 24	12 out 24	No *p* value, have SMDg ¼ 0.38 between groups	Significant reduction in depression in BReF intervention group compared to baseline	Small sample size, nonresponse bias due to 17% response rate
Shirazian et al.^33^	2023	USA	PHQ-9	RCT (Behavioral education intervention) versus (control group on education) for 12 weeks and eight sessions. Outcomes were measured at weeks 0, 8, and 16	23 out 45	22 out 45	*p* > 0.05 between groups	No significant reduction in depression despite participants’ positive view of the intervention	Turnover in social workers performing the intervention, unequal drop-out rates. Limited social worker training
Rahimipour et al.^34^	2015	Iran	DASS-21	RCT (hope therapy) versus (placebo). Hope therapy, lasting 60–90 min, was administered weekly during dialysis sessions for 8 weeks	25 out 50	25 out 50	*p* < 0.05 between groups	Significant reduction in depression in hope therapy group compared to baseline. Significant reduction in depression is greater in hope therapy group compared to control	Small sample size
Bennett et al.^35^	2020	USA	PHQ-4	RCT (laughter therapy session) versus (no intervention (control)), 30 min weekly group laughter therapy session for 8 weeks	70 out 151	72 out 151	*p* = 0.05 between group	Significant reduction in depressive symptoms in the intervention group compared to baseline	Baseline differences between the control and intervention groups could not be analyzed, adjustment for these differences not possible due to a high rate of incompletion
Nassim et al.^36^	2021	Canada	PHQ-9	RCT (BMI) versus (control group called HEP) over 8 weeks	30 out 60	30 out 60	Pre–post intervention group: *p* < 0.001 and *p* = 0.62 between groups	Significant reduction of depression scores in both intervention and control group compared to baseline. No significant reduction between intervention and control	Facilitation of both programs by the same interventionist
Rigas et al.^37^	2022	Canada	PHQ-9	RCT (BMI) versus (control, HEP) Reevaluated 6 months of follow-up data from an 8 week RCT that assessed the effects in hemodialysis patients with depression and/or anxiety symptoms	30 out 55	25 out 55	Pre–post intervention group: *p* = 0.008 and *p* = 0.650 between groups	Significant reduction for depression scores in both BMI and HEP group compared to baseline. No significant reduction between groups	Modest sample size, lack of waitlist control
Thomas et al.^38^	2017	Canada	PHQ-9	RCT (meditation) versus (no intervention). For 8 weeks, 10−15 min individual chairside meditation session three times a week during hemodialysis	21 out 41	20 out 41	*p* = 0.45 between groups	No significant reduction in depression scores despite mindfulness being both feasible and well-tolerated by hemodialysis patients	Small sample size
Shokrpour et al.^39^	2021	Iran	DASS-21	RCT (positive thinking skills through eight workshop sessions) versus (control group (no intervention))	35 out 70	35 out 70	*p* > 0.05 between groups	No significant reduction in depression in both control and intervention groups compared to baseline	Self-reporting tools, limitation of physical conditions of patients to attend training classes for extended period
González-Flores et al.^40^	2023	Mexico	BDI	RCT (control group (cognitive behavioral strategies)) versus (experimental group (same strategies plus resilience model)). Outcomes were assessed in the beginning, 8 weeks, and 4 weeks after treatment	25 out 53	28 out 53	*p* = 0.02 between groups	Significant reduction in depression for both groups compared to baseline	No reinforcement sessions for intervention group compared to control
Nadort et al.^41^	2022	Netherlands	BDI-II	RCT (online self-help problem-solving group) versus (standard care group). The intervention, “Worry Less for Dialysis Patients,” contained five sessions with information, analogy, and homework to be completed over 10 weeks	101 out 190	90 out 190	*p* = 0.94 between groups	No significant reduction in depression between groups despite decrease in BDI-II scores compared to baseline	High dropout rate, nonadherence
Dingwall et al.^42^	2021	Australia	PHQ-9	RCT (Immediate treatment with the Stay Strong (ISS) app versus HepB Story app, followed by Stay Strong (HepB/DSS) at 3 months versus Treatment as Usual with DSS app (TAU/DSS; control) introduced at 3 months) in a 2:2:1 ratio. Follow-up assessments were conducted at 3 and 6 months	33 out 156	Stay Strong app: 62 out 156 HepB/DSS app: 61 out 156	TAU/DSS versus HepB/DSS: *p* = 0.03 ISS versus TAU/DSS: *p* = 0.31 ISS versus HepB/DSS: *p* = 0.17	HepB Story revealed significant reductions in PHQ-9 scores at 3 months, but not for Stay Strong or TAU. No significant differences in PHQ-9 scores were found comparing the groups	Small sample size, lack of control group without use of app
Griva et al.^43^	2018	Singapore	HADS	RCT (the HED-SMART group) versus (standard care group). The intervention, grounded in social cognitive theory, focused on enhancing patients’ self-management capabilities for improved disease management during hemodialysis	134 out 235	101 out 235	*p* = 0.03 between group	Significant reduction in depressive symptoms between groups	Convenience sample, possibility for self-selection bias
Igarashi et al.^44^	2022	Brazil	BDI	Prospective interventional study; followed a short-term meditation protocol during each hemodialysis session, lasting 10−20 min, three times weekly for 12 weeks	None	22	Pre–post *p* = 0.014	Significant reduction in depressive symptoms	Small sample size
Music therapy									
Imani et al.^45^	2021	Iran	BDI	RCT (Instrumental music intervention) versus (routine care) for 3 weeks, with 20-min sessions held three times a week	25 out 50	25 out 50	*p* = 0.001 between groups	Significant reduction in depression in the intervention group compared to baseline. Significant reduction in depression between groups	Use of self-report tools, nonuse of indicators related to the main disease, poor cooperation from staff, patient impatience with questionnaire, interruption of music due to manipulation of headphones
Bro et al.^46^	2022	Denmark	HADS	RCT (live music) versus (routine care) Two clusters of 12 patients were randomized and 30 min of live music weekly were received during hemodialysis for 6 weeks	12 out 24	12 out 24	*p* = 0.406 between groups	No significant reduction in depression between groups	Pilot trial, no blinding, self-reported measurements
Burrai et al.^47^	2019	Italy	HADS	RCT (live singing) versus (standard care), 15 min of live singing during six consecutive hemodialysis sessions while the other underwent standard care. After a washout period of 2 days, the two groups switched interventions	12 out 24	12 out 24	*p* < 0.001 between groups	Significant reduction in depression scores between groups	Pilot study, small sample size
Hagemann et al.^48^	2019	Brazil	BDI-II	Prospective interventional study; patients attended a total of eight music therapy sessions, scheduled twice a week with each session lasting 75 min. QOL and depressive symptoms were evaluated before and after intervention	None	23	Pre–post *p* < 0.001	Significant reduction in depression symptoms	Lack of control group

BDI-II: Beck’s Depression Inventory-II; BMI: Brief Mindfulness Intervention; BReF: Cognitive Cehavioural Therapy for Renal Fatigue; CBT: cognitive behavioral therapy; CESDS/CES-D: Center for Epidemiologic Studies Depression Scale; CI: Confidence Interval; DASS-21, Depression Anxiety Stress Scale-21; DSI: Depressive Severity Index; DSS: Delayed Stay Strong; HADS-D: Hospital Anxiety and Depression Scale-Depression; HED-SMART: HEmoDialysis Self-Management Randomized Trial; HEP: Health Enhancement Program; HepB: Hepatitis B; MADRS: Montgomery–Asberg Depression Rating Scale; PHQ-9: Patient Health Questionnaire-9; QOL: Quality of Life; RCT: randomized controlled trial; QIDS-C: Quick Inventory of Depressive Symptomatology–Clinician rating; SMDg, standardized mean difference; TAU: Treatment As Usual; VR: Virtual Reality; ZSDS: Zung’s Self-Rating Depression Scale.

### Assessment of risk of bias and quality of studies

As recommended by the Cochrane Collaboration, we analyzed the bias and quality in RCTs based on the Risk of Bias 2 (RoB 2) tool.^17^ The RoB 2 tool evaluates five key areas: randomization procedures, deviations from the intended intervention, missing outcome data, outcome measurement, and the selection of reported results.^17^ We also considered potential biases related to outcome assessment and selection in the published findings. Each study was carefully reviewed and given a score on the risk scale of “low,” “some,” or “high” based on these criteria.

For non-randomized studies, the Risk of Bias in Non-randomized Studies of Interventions (ROBINS-I) tool has been the standard, as recommended by the Cochrane Collaboration.^18^ The ROBINS-I tool assesses bias arising from confounding, selection, classification of interventions, deviations from the intended intervention, missing data, outcome measurement, and the selection of reported results.^18^ Each non-randomized study was given a score of “low,” “moderate,” or “serious.” Two independent reviewers (A.C. and S.S.) conducted the RoB 2 and ROBINS-I assessments, with any disagreements resolved through discussion.

## Results

### Study selection

**Figure 1.**
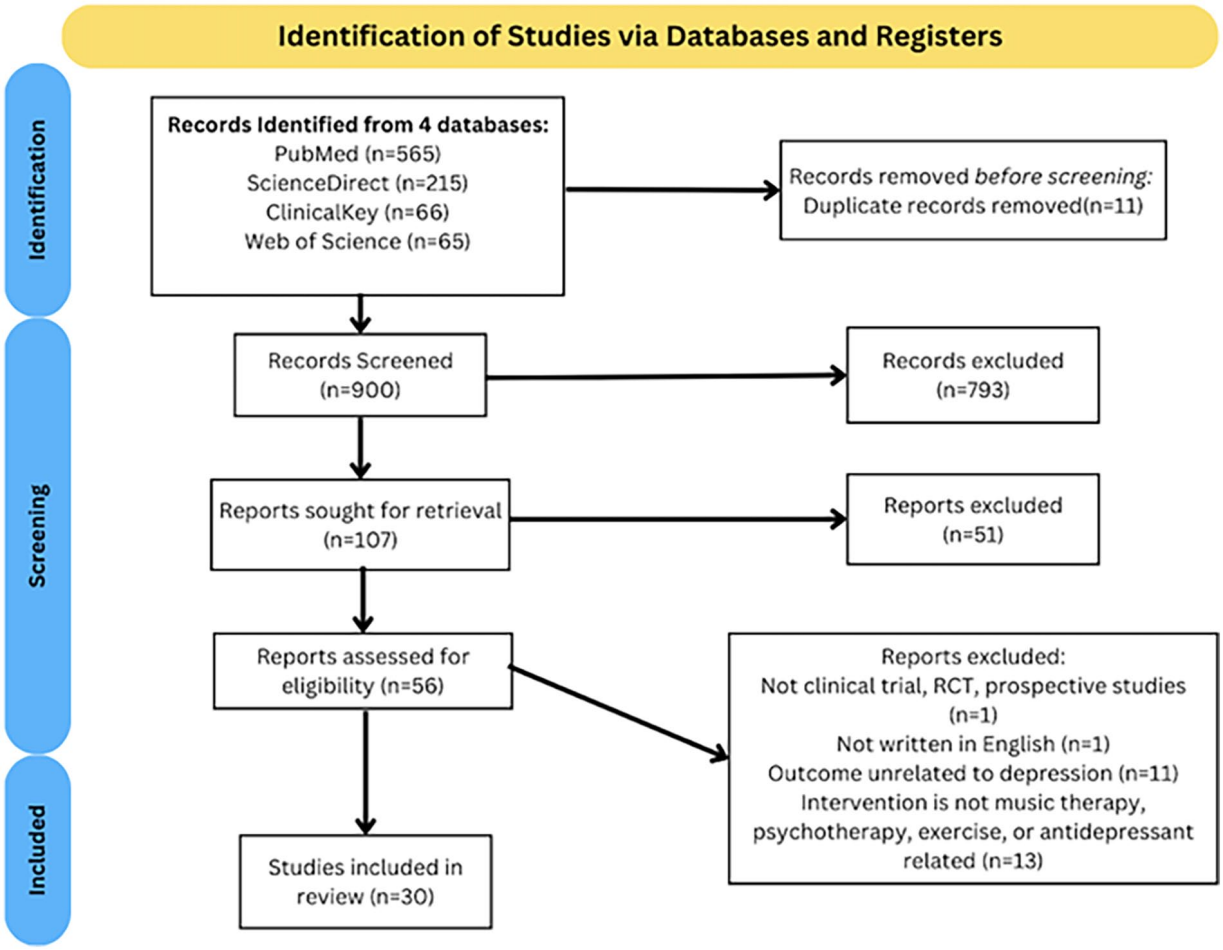
PRISMA flow diagram for selection process. PRISMA: Preferred Reporting Items for Systematic Reviews and Meta-Analysis.

Out of 911 records that were identified, 900 were screened for their title and abstract. After evaluating 56 full-text articles for eligibility, 30 studies were selected for qualitative synthesis. Figure 1, the flowchart using the PRISMA guideline, is shown below.^15,16^

### Study characteristics

The characteristics of the 30 included studies and their outcomes are summarized in Table 1. Four studies measured the efficacy of antidepressants, including fluoxetine, sertraline, and a variety of other antidepressants.^19–22^ One study directly compared the efficacy of sertraline versus CBT.^19^ Nine studies assessed the impact of different types of exercise on dialysis patients.^23–31^ Six studies focused on intradialytic exercise, which involves aerobic and non-aerobic workout regimens performed during dialysis sessions.^24–26,29–31^ One study investigated the effects of walking therapy.^27^ Others consisted of yoga and resistance therapy.^23,28^ Thirteen studies explored various psychotherapy methods, including three on CBT, one on positive thinking, one on self-help, one on in-app therapy, one on hope, one on laughter, and five on meditation.^32–44^ Additionally, four studies investigated the effects of music therapy.^45–48^ In total, there were 22 RCTs, 1 nonrandomized controlled trial, 6 prospective interventional studies, and 1 prospective cohort study. Two out of 30 studies, Nassim et al. and Sheshadri et al., included patients on different types of dialysis, while the remaining studies focused exclusively on chronic hemodialysis patients.^27,36^

Various types of depression scales were used such as Patient Health Questionnaire-9 (PHQ-9), Beck’s Depression Inventory-II (BDI-II), Montgomery–Asberg Depression Rating Scale (MADRS), Quick Inventory of Depressive Symptomatology–Clinician rating (QIDS-C), Center for Epidemiologic Studies Depression Scale (CES-D), Zung’s Self-Rating Depression Scale, Hospital Anxiety and Depression Scale, and Depression Anxiety Stress Scale-21. Detailed information on treatment and control characteristics can be found in Table 1.

### Results of the studies

#### Antidepressant studies

Mehrotra et al. examined treatment outcomes by comparing sertraline and CBT over 12 weeks.^19,21^ Both interventions decreased QIDS-C scores, but sertraline was associated with a significantly greater reduction in depressive symptoms compared to CBT (*p* = 0.035).^19^ Friedli et al.’s RCT reported significant reductions in BDI-II and MADRS scores for both the sertraline and control (placebo) groups, though there was no significant difference (*p* = 0.2) between them.^20^ Kauffman et al. also demonstrated a significant decline in PHQ-9 scores following 12 weeks of fluoxetine treatment (*p* = 0.002), with 14 out of 16 participants no longer meeting the criteria for MDD (*p* < 0.001), although the absence of a comparison group limits the strength of these findings.^21^ On the other hand, Guirguis et al.’s study found that 22 out of 30 patients with initial BDI-II scores above 16 continued to show elevated scores, which the authors attributed to inconsistent medication review and improper dosing of antidepressants.^22^ Additionally, six participants had no prior or ongoing diagnosis of MDD throughout the study.^22^

#### Exercise studies

Among the RCTs, most studies reported no significant between-group differences in depressive symptoms. Nakamura-Taira et al. compared resistance training with stretching as a control group and found no significance.^23^ Similarly, the walking intervention by Sheshadri et al. found no significant difference in depressive symptom outcomes between the intervention and standard care at 3 months (*p* > 0.05).^27^ While both Sheshadri et al. and Nakamura-Taira et al. conducted RCTs, it is important to note that the participants in Sheshadri et al.’s study had both hemodialysis and peritoneal dialysis patients, which may have introduced variability that affected outcomes.^23,27^ In the study by Turoń-Skrzypińska et al., BDI-II scores significantly decreased after 3 months of low-intensity virtual reality cycling while depressive symptoms worsened in the control group which received no intervention; yet, the between-group difference was not significant (*p* = 0.2).^25^ Similarly, Kauric Klein, reported a significant within-group reduction in depressive symptoms in the yoga group over 12 weeks, while the standard care group showed no significant change, though between-group differences were not statistically significant.^28^

Among the remaining RCTs, two studies compared two active exercise interventions and reported significant reductions in depression symptoms within the intervention groups; however, between-group differences were not statistically significant.^24,26^ Zhou et al. conducted a study using a virtually supervised exercise program versus nursing supervised group and found significant reductions in CES-D scores in both groups after the intervention (*p* < 0.001), with no significant difference between them (*p* = 0.246).^24^ Similarly, Ortega-Pérez de Villar et al. compared intradialytic exercise with a home-based exercise program and reported decrease in depression scores in both groups (*p* = 0.017), again with no significant difference between the interventions (*p* = 0.081).^26^

In addition, three prospective interventional studies showed significant downtrend of depressive symptoms after treatment (*p* < 0.01), although the absence of control groups limits the generalizability of their findings.^29–31^

#### Psychotherapy studies

Significant decreases in depressive symptoms were observed in the CBT-based resilience intervention compared to standard cognitive behavioral strategies, as reported by González-Flores et al. (*p* = 0.02).^40^ Picariello et al. noted a small to moderate between-group effect size (standardized mean difference = 0.38) when comparing a CBT-fatigue intervention to a waitlist group; however, no *p* value was provided, limiting interpretation of statistical significance.^32^ Both hope therapy (*p* < 0.05) and laughter therapy (*p* = 0.05) demonstrated significant decreases in depressive symptoms compared to no intervention groups.^34,35^

Findings from mindfulness-based interventions were mixed across five studies. Nassim et al. and Rigas et al. observed decreases in depressive symptoms in both Brief Mindfulness Intervention (BMI) group and the control, Health Enhancement Program (HEP), group, though no significant between-group differences were identified (*p* = 0.62).^36,37^ In contrast, Griva et al. found a significant between-group difference which showed that self-management intervention incorporating mindfulness decreased depressive symptoms more than standard care (*p* = 0.03).^43^ Nevertheless, Thomas et al. found no significant differences between mindfulness and control groups (*p* = 0.45).^38^ Igarashi et al., in a prospective study without a control group, reported a significant pre–post decrease in depressive symptoms (*p* = 0.014).^44^

In contrast to these interventions, Shirazian et al. (*p* > 0.05), Shokrpour et al. (*p* = 0.465), Nadort et al. (*p* = 0.94), and Dingwall et al. (*p* > 0.05) all reported non-significant between-group differences for a behavioral education or self-help intervention, though some observed pre–post improvements within group.^33,39,41,42^

#### Music therapy studies

Four articles studied the effects of music therapy primarily during intradialytic sessions, occurring two to three times per week and lasting from 20 to 75 min.^45–48^ Imani et al. and Burrai et al. reported significant differences in between the music therapy group and routine care (*p* = 0.001 and *p* < 0.001, respectively).^45,47^ However, Bro et al. found no significant between-group effects for depression (*p* = 0.406).^46,47^ Notably, Bro et al. did find a significant reduction in levels of anxiety (*p* = 0.011).^46^ Hagemann et al., the only prospective interventional study, observed a significant reduction in depression symptoms following eight sessions of music therapy (*p* < 0.001).^48^

## Discussion

MDD and depressive symptoms are prevalent among patients undergoing dialysis. Many patients experience fatigue, a common side effect of dialysis, which can be misinterpreted as depression due to the overlap in symptoms. Fatigue, decreased appetite, energy, motivation, and weight loss are symptoms shared by both depression and dialysis.^49^ Financial constraints, decreased appetite, increased fatigue, combined with the challenges of managing a chronic illness, often lead to diminished quality of life and worsened depressive symptoms.^2–4,6–8^ These factors can reduce motivation for self-care and adherence to treatment, resulting in increased hospitalizations and, in severe cases, suicidal ideation.^7,8^ Consequently, effective depression interventions tailored for this population are crucial for patient-centered care.

### Antidepressants

Second-generation antidepressants with psychotherapy have traditionally been the first-line treatment for moderate to severe MDD, including dialysis patients.^9^ Similarly, a Cochrane Library’s 2016 review has suggested that shorter-term SSRI use may lower clinical depression scores compared to placebo, though the quality of evidence was ungradable.^10^ However, our reviews have raised safety concerns regarding their use in this demographic. Increased pill burden and side effects can deter patients from adhering to these treatments.^20^ Due to limited data, the impact of SSRIs on all-cause mortality; suicide; adverse effects including as dizziness, hypotension, headache, and sexual dysfunction; withdrawal from dialysis; and hospitalization, have not been well studied. The Cochrane Library’s 2016 review found no statistically significant difference between SSRIs and group psychological training in reducing depressive symptoms, and treatment discontinuation was difficult to assess due to the low number of reported cases.^10^ However, a more recent study by Mehrotra et al. demonstrates that sertraline led to greater reductions in depression scores at 12 weeks than CBT, though it was associated with a higher incidence of adverse events.^19^

Moreover, our systematic review indicates that the effectiveness of risks and benefits discussion of antidepressants for dialysis patients remains uncertain, primarily due to polypharmacy and adverse effects.^22^ Kauffman et al. showed that fluoxetine demonstrated an 80% remission rate for depressive symptoms but also reported one patient who discontinued the medication due to severe side effects, including abdominal cramping, dizziness, vomiting, and lightheadedness.^21^ Other patients experienced milder effects such as nausea, headaches, drowsiness, and agitation, particularly when taken without food.^21^ Guirguis et al. highlight nephrologists’ hesitancy to prescribe these medications, citing the complexities of managing multiple coexisting conditions and extensive medication regimens, which increase the likelihood of drug interactions.^22^ For instance, citalopram is generally avoided due to serious cardiac complications, including dose-dependent QT prolongation, and the significant concern of coronary artery disease-related deaths in ESRD patients.^22^ Additionally, Friedli et al. found that nearly a quarter of participants encountered adverse effects such as nausea and infections, with some classified as serious, including a fatal case potentially linked to the medication.^20^

While SSRIs, particularly sertraline and fluoxetine, have shown potential benefits in improving depressive symptoms in dialysis patients, the overall evidence remains mixed, with consistent concerns about adverse effects, medication interactions, and study quality. Given the high risk of polypharmacy and the burden of comorbid conditions in this population, a routine or universal recommendation of antidepressants cannot be made at this time. Instead, prescribing antidepressants should be approached on a case-by-case basis, incorporating multidisciplinary input, especially from psychiatry and nephrology, and close monitoring for efficacy and tolerability. Future research should prioritize larger randomized trials with longer follow-up periods and standardized psychiatric assessments to clarify who may benefit most from pharmacologic treatment.

### Exercise

Intradialytic exercise, whether virtual, supervised, or group-based, displays potential in alleviating depressive symptoms with fewer side effects. A 2019 Cochrane Library review on psychosocial interventions in dialysis patients found that exercise interventions may help lower the risk of both MDD (Relative Risk (RR) 0.47, 95% CI, 0.27–0.81) and contribute to improvements in quality of life (mean difference (MD) 3.06, 95% CI, 2.29–3.83), though evidence range was low to moderate.^11^ A key advantage of these programs is that they optimize the time patients already spend in dialysis center, reducing the need for additional commitments. They also provide interactive, professionally supervised sessions that can enhance emotional well-being and break the monotony of routine dialysis. More recently, Zhou et al. demonstrated that a remotely monitored, individualized exercise intervention achieved benefits similar to nurse-supervised therapy in patients with severe depression, without placing additional responsibility on healthcare staff.^24^ Some studies, however, report mixed findings regarding the superiority of intradialytic over home-based exercise. For example, the walking study by Sheshadri et al. and resistance home-based exercises by Nakamura-Taira et al. indicate that home exercises were less effective in reducing depressive symptoms.^23,27^ In contrast, Ortega-Pérez de Villar et al. found no significant difference in depression or physical functioning when comparing intradialytic with home-based exercise programs.^26^ In addition, intradialytic yoga did not significantly improve depressive symptoms but was associated with positive impact on blood pressure and anxiety.^28^ These findings suggest that while intradialytic exercise hold promise for improving mental health in dialysis patients, its effectiveness may vary across different modalities. Further research is needed to develop tailored approaches that address individual patient needs that optimize treatment outcomes.

### Psychotherapy

Given that the risks and benefits of antidepressants for treating depression in dialysis patients remain inconclusive, there has been a growing focus on exploring the effectiveness of psychosocial interventions for managing depression in this population. The 2019 Cochrane Library review, which included 22 studies and 2,056 participants, found that CBT likely improves depressive symptoms (MD −6.10, 95% CI, −8.63 to −3.57).^11^ The review also suggests counseling has a modest effect on depressive symptoms (MD −3.84, 95% CI, −6.14 to −1.53).^11^ Spiritual practices show inconsistent effects, and there are limited data on acupressure, telephone support, and meditation.^11^

Psychotherapy, particularly CBT, is widely recognized as an effective and one of the first-line treatments for depression, supported by extensive research across various populations.^9^ However, despite its proven efficacy in general settings and positive results from the Cochrane Library review as stated above, our review of CBT in dialysis patients continues to yield inconclusive results, as shown in Table 1. When analyzing the qualitative interviews performed during the study, it indicated that while CBT during dialysis sessions can make the time pass more quickly, fatigue and drowsiness often hinder participation.^33^ Nevertheless, privacy concerns during treatment sessions further complicate implementation. Research by Picariello et al. highlights that shift in negative perceptions of fatigue, rather than changes in anxiety and depression, are key mediators in reducing fatigue severity.^32^

Emerging interventions such as hope therapy aim to enhance patients’ sense of optimism, purpose, and resilience in facing chronic conditions. This approach equips patients with psychological tools to achieve realistic and meaningful goals, cope with setbacks, and engage in group therapies, fostering a more positive and resilient mindset.^34^ Similarly, laughter therapy, which focuses on inducing genuine laughter, has been linked to psychological and physiological benefits.^35^ This therapy has been well-received by patients, and group sessions foster a sense of community and shared experience, contributing to overall positive outcomes. Studies suggest that integrating laughter therapy into routine care for dialysis patients may enhance their mental health and overall well-being.^35^

According to our review, mindfulness interventions, including brief meditation programs, have also shown potential in decreasing depression and anxiety symptoms among patients in dialysis. Some studies comparing BMI to active controls like HEP found that both groups reduced symptoms of depression, with no differences in between groups, but BMI was more effective in reducing anxiety symptoms.^36,37^ Other studies showed mixed or inconclusive findings.^38,43,44^ Overall, mindfulness techniques appear feasible and potentially beneficial, particularly for anxiety management, but further research is needed to clarify their comparative efficacy and long-term effects.

Other studies have revealed that combining CBT with the resilience model and the Wellbeing Intervention for Chronic Kidney Disease, an approach integrating educational, behavioral, and supportive elements, led to significant improvements in depressive symptoms, anxiety, and quality of life among patients on chronic hemodialysis.^40,42^ In contrast, positive thinking training and internet-based treatment had mixed results.^39,41^ Overall, psychotherapy interventions that prioritize fatigue management, foster resilience, and incorporate relaxation or mindfulness techniques show the most promise in addressing the unique mental and physical setbacks faced by dialysis patients, offering a pathway to healthier mentality and quality of life.

### Music

Music therapy, a more accessible method of relaxation, particularly in inpatient settings, has not always been studied for depression management. However, the effectiveness of music in enhancing emotional well-being has been well-documented.^50^ The healing art of music therapy has shown benefits for patients in acute hospital settings.^51^ Three out of the four studies revealed significant decreases in depressive symptoms.^45,47,48^ Although Bro et al. did not report significant differences in depressive symptoms before and after the intervention, they noted a significant reduction in anxiety levels.^46^ Hagemann et al. found that patients receiving dialysis nearby, though not directly participating in the study, were positively engaged in the music therapy and indirectly benefited from it.^48^ These findings suggest that while music therapy has proven benefits for emotional well-being and may help reduce depressive symptoms in dialysis patients, its effectiveness can vary. Further exploration is needed to refine its application and understand its broader impact, including potential benefits for patients not directly involved in the therapy.

Incorporating intradialytic exercise, music therapy, and psychotherapeutic interventions targeting resilience and fatigue management offers promising benefits in improving patient satisfaction and reducing depression. These therapies effectively utilize the dialysis session as an opportunity for both physical and mental health support, enhancing overall well-being while addressing the unique challenges faced by dialysis patients.

## Limitations

**Figure 2.**
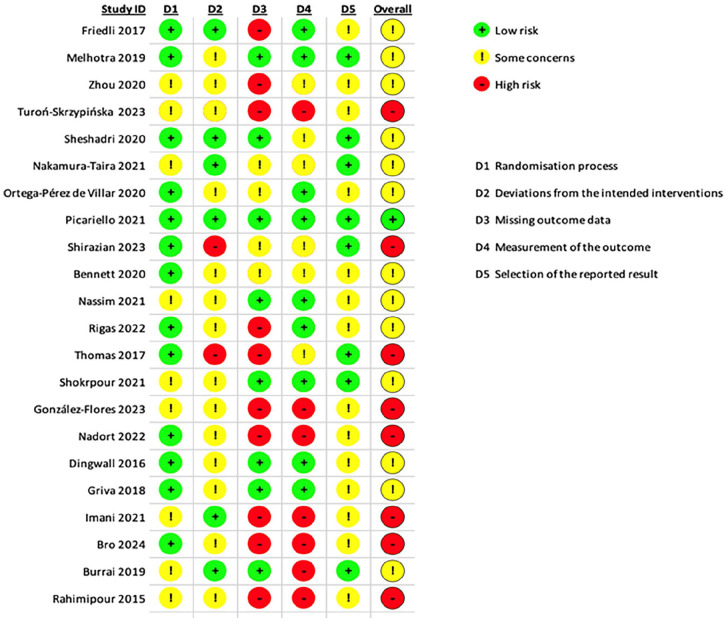
RoB 2 tool results. RoB 2: Risk of Bias 2.

**Figure 3.**
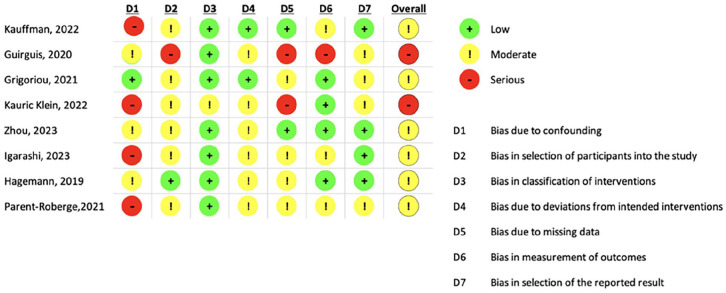
ROBINS-I tool results. ROBINS-I: Risk of Bias in Non-randomized Studies of Interventions.

Several studies examining interventions for depression in dialysis patients share common limitations that may impact the interpretation and generalizability of their findings as seen in Figures 2 and 3. Numerous studies are limited by small sample sizes, which often do not adequately represent the broader dialysis population, particularly regarding gender and racial diversity. Many of the studies included participants who screened positive for MDD based on depression scales but did not have a prior MDD diagnosis, which could be a limitation. Unfortunately, most chronic kidney disease and ESRD patients do not receive a formal MDD diagnosis or treatment due to the overlapping symptoms of post-dialysis effects and depression.^14,22^ Additionally, the absence of control groups in many studies raises concerns about the validity of conclusions, as participants often serve as their own controls without the comparison to a non-intervention group. Short treatment durations also pose a challenge, given that chronic depressive episodes can last much longer than the periods studied, making it difficult to assess the long-term efficacy of interventions. Recruitment difficulties, particularly during the COVID-19 pandemic, have led to high dropout rates, which introduced bias and hindered the reliability of results. Furthermore, many patients in chronic hemodialysis are elderly and functionally impaired, complicating their responsiveness to treatment and participation in interventions.^22^ Single-center designs are also prevalent, restricting the generalizability of findings to other settings, while reliance on self-reported measures can introduce biases that affect the accuracy of the data collected. Lastly, variations in intervention exposure and a lack of randomization in several studies may further confound the results.

One methodological limitation is the restriction of the literature search to studies published between 2017 and 2023. Although this timeframe was selected to emphasize recent advances, it may have excluded earlier primary studies, potentially introducing selection bias and limiting the context of our findings. Notably, Rahimipour et al., published in 2015, was retrieved during the original search despite the date limit, likely due to indexing discrepancies. Given its unique focus on hope therapy, an intervention underrepresented in more recent literature, the study was included based on its conceptual relevance and adherence to other eligibility criteria. This deviation from the predefined search window is transparently noted in the narrative synthesis and clearly documented in the methodology (Supplemental Appendix 2). While this exception may introduce bias, it was made to preserve the comprehensiveness and relevance of the review. Furthermore, despite exhaustive search efforts, relevant studies may have been missed, particularly unpublished works, those indexed in less accessible databases, or publications in languages other than English. These inherent limitations highlight the necessity for ongoing literature surveillance to capture emerging evidence as the field evolves.

Furthermore, the heterogeneity of the studies, including the use of various depression assessment tools, makes it difficult to draw direct comparisons across studies. Collectively, these limitations underscore the need for more robust, multicenter studies with bigger and more inclusive sample sizes, as well as improvements in the review methodology, to validate and expand upon the current findings.

## Conclusion

This study explores various interventions for managing depression in hemodialysis patients and highlights both promising strategies and significant challenges. Despite the potential benefits of approaches such as antidepressants, intradialytic exercise, music therapy, and psychotherapy, the limitations of the current studies, including small sample sizes, lack of control groups, short treatment durations, and recruitment challenges, hinder the ability to draw definitive conclusions. The predominance of elderly and functionally impaired participants complicates treatment responsiveness and adds another layer of difficulty in generalizing findings to the wider hemodialysis population.

Furthermore, the absence of diverse patient representation raises questions about the applicability of results across different demographic groups. While antidepressants have been more extensively studies, the findings are mixed, and concerns about safety, polypharmacy, and adherence require a careful, individualized approach. Non-pharmacological interventions show promise and remain as an important avenue for further investigation, particularly in patients where antidepressants may not be appropriate. Given these challenges, future research should prioritize multicenter trials with larger, more diverse populations and robust designs that include control groups to better assess the efficacy of interventions. Addressing these limitations will enhance our understanding of effective strategies for improving mental health outcomes in this vulnerable patient population, ultimately contributing to more effective, patient-centered care.

## Supplemental Material

sj-docx-1-smo-10.1177_20503121251353028 – Supplemental material for A comprehensive systematic review of pharmacological and non-pharmacological depression interventions for patients on dialysis Supplemental material, sj-docx-1-smo-10.1177_20503121251353028 for A comprehensive systematic review of pharmacological and non-pharmacological depression interventions for patients on dialysis by Ahyeon Cho, Tammy Tran, Laura Telfer, Ahmad Matarneh, Sundus Sardar and Nasrollah Ghahramani in SAGE Open Medicine

sj-docx-2-smo-10.1177_20503121251353028 – Supplemental material for A comprehensive systematic review of pharmacological and non-pharmacological depression interventions for patients on dialysis Supplemental material, sj-docx-2-smo-10.1177_20503121251353028 for A comprehensive systematic review of pharmacological and non-pharmacological depression interventions for patients on dialysis by Ahyeon Cho, Tammy Tran, Laura Telfer, Ahmad Matarneh, Sundus Sardar and Nasrollah Ghahramani in SAGE Open Medicine
