# Supplementary material for: A comprehensive systematic review of pharmacological and non-pharmacological depression interventions for patients on dialysis
Source: SAGE Open Med. 2025 Aug 23;13:20503121251353028. doi: 10.1177/20503121251353028 (PMC12375159; doi:10.1177/20503121251353028)
Supplement: sj-docx-2-smo-10.1177_20503121251353028 – Supplemental material for A comprehensive systematic review of pharmacological and non-pharmacological depression interventions for patients on dialysis [file sj-docx-2-smo-10.1177_20503121251353028.docx]

# Appendix 1. Electronic Search Strategies

*Note: *MeSH* = Medical Subject Headings; *tiab* = title/abstract; * (asterisk) = truncation symbol used to retrieve word variants (e.g., antidepressant, antidepressants).*

## PubMed (MEDLINE)

1. "Depression"[MeSH]
2. "Depressive Disorder"[MeSH]
3. "Major Depressive Disorder"[tiab] OR depress*[tiab] OR MDD[tiab] OR depressive disorder[tiab]
4. #1 OR #2 OR #3
5. "Renal Dialysis"[MeSH]
6. dialysis[tiab] OR hemodialysis[tiab] OR peritoneal dialysis[tiab] OR ESRD[tiab]
7. #5 OR #6
8. "Depression treatment"[tiab] OR "Depression intervention"[tiab] OR SSRI[tiab] OR antidepressant*[tiab] OR psychosocial intervention*[tiab] OR psychotherapy[tiab] OR CBT[tiab] OR "cognitive behavioral therapy"[tiab] OR exercise[tiab] OR physical activity[tiab] OR relaxation[tiab] OR meditation[tiab]
9. #4 AND #7 AND #8

Filters applied: English, Humans, Full-text available, Article types (RCTs, Clinical Trials), Publication date from 01/01/2017 to 12/31/2023.

## ScienceDirect

1. "MDD" OR "Depression" OR "Depressive Disorder"
2. "Dialysis" OR "Hemodialysis"
3. "Depression Treatment" OR "Depression Intervention" OR SSRI OR antidepressant* OR psychosocial intervention* OR psychotherapy OR CBT OR exercise OR relaxation OR meditation
4. #1 AND #2 AND #3

Filters applied: English, Full-text, Research articles (RCTs and clinical trials), Publication date 2017–2023.

## ClinicalKey

1. "Depression" OR "Depressive Disorder"
2. "Dialysis" OR "Hemodialysis"
3. "Depression Intervention" OR "Depression Treatment" OR SSRI OR antidepressant* OR psychosocial intervention* OR psychotherapy OR CBT OR exercise OR relaxation OR meditation
4. #1 AND #2 AND #3

Filters applied: English, Full-text, Research articles, RCTs and clinical trials, Publication date 2017–2023.

## Web of Science

1. "MDD" OR "Depression" OR "Depressive Disorder"
2. "Dialysis" OR "Hemodialysis"
3. "Depression Intervention" OR "Depression Treatment" OR SSRI OR antidepressant* OR psychosocial intervention* OR psychotherapy OR CBT OR exercise OR relaxation OR meditation
4. #1 AND #2 AND #3

Filters applied: English, Article type (RCTs, Clinical Trials), Full-text (if available), Publication date 2017–2023.

## Reference Lists (Hand-searching)

Reference lists were manually reviewed to identify additional eligible studies. All included studies, including Rahimipour et al. (2016), were retrieved through the formal database searches. The inclusion of Rahimipour et al. despite being outside the 2017–2023 date range is due to indexing inconsistencies.

**Appendix 2: Table 2 Baseline Characteristics and Demographics of Study Participants**

**Antidepressants**

| **First Author (Reference)** | **Mean Age (SD)** | **Gender** | **Ethnicity** | **Depression** |
| --- | --- | --- | --- | --- |
| **Kauffman (21)** | 56.7 (16.2) | Female: 13 (81%)  Male: 3 (19%) | African American: 13 (81%), White: 3 (19%) | All patients with untreated Major Depression Disorder |
| **Guirguis(22)** | 62 (16) | Male (63%) | White (73%) | BDI-II: 26 (IQR 19) Moderate Depression  PHQ-9: 12 (IQR 9) Moderate Depression |
| **Friedli (20)** | 59 (13.8) | Male (77%) | White (60%, Asian (20%),  Other (20%) | BDI-II: 29.1 ± 8.4 Severe Depression  MADRS: 24.9 ± 4.3 Moderate Depression |
| **Mehrotra(19)** | 51 (13) | Male (57%) | White (43%), Black (28%), Native American (8%),  Hispanic (28%), Other (21%) | Control BDI-II: 26.2 (95% CI: 23.6 to 28.8) Moderate Depression    Intervention BDI-II: 25.8 (95% CI: 23.3 to 28.4) Moderate Depression |

**Exercise**

| **First Author (Reference)** | **Mean Age (SD)** | **Gender** | **Ethnicity** | **Depression** |
| --- | --- | --- | --- | --- |
| **Zhou** (24) | Control: 66.5 (10.0)  Intervention: 62.7 (6.8) | Control: Female (49%)  Intervention: Female (61%) |  | At risk for clinical depression based on CES-D ≥ 16  Control: 16 (44%) Possible Depression  Intervention: 11 (30%) Unlikely Depression |
| **Turoń-Skrzypińska**  (25) | Control: 62.63 (15.47)  Intervention: 57.56 (17.61) | Control: Male (63%)  Intervention: Male (74%) |  | Control BDI-II: 9.09 ± 8.25  Minimal Depresision  Intervention BDI-II: 9.53 ± 6.43 Minimal Depression  Depression diagnosis not a requirement for inclusion. |
| **Grigoriou(29)** | 59 (13.7) | Male: 16 (80%)  Female; 4 (20%) |  | ZSDS: 41.3 ± 6.0 Minimal Depression  BDI: 5.00 ± 4.8  Minimal Depression |
| **Sheshadri (27)** | Control: 56  Intervention: 60 | Control: Male (63%)  Intervention: Male (93%) | Control: Hispanic (17%), White (17%), Black (37%), Asian (20%), Native Hawaiian/Pacific Islander (10%), Other (10%)    Intervention: Hispanic (17%), White (13%), Black (47%), Asian (20%), Native Hawaiian/Pacific Islander (7%), Other (14%) | CESD: 7.9 ±7.3 Unlikely Depression |
| **Kauric-Klein(28)** | 65.32 (14.43) | Male (52.6%) | White (63.1%), African American (36.8%) | PHQ-9: 4.26 ± 3.02 Minimal to Mild Depression |
| **Parent-Roberge (30)** | 73 | Female: 9 (53%) |  | BDI: 4.5 (1.8-6.0) Minimal Depression |
| **Zhou (31)** | 55.03 (10.69) | Male: 35 (46.7%)  Female: 40 (53.3%) |  | HADS-D: 9.09 ± 4.08 Mild Depression |
| **Nakamura-Taira**  (23) | Control: 72.57 (2.26)  Intervention: 74.90 (2.23) | Control: Male 15 (71.43%)  Female 6 (28.57%)    Intervention: Male 7 (33.33%)  Female 14 (66.67%) |  | PHQ-9: 5.93 ± 0.72 Minimal Depression |
| **Ortega-Pérez de Villar**  (26) | Control: 59.3 (16.1)  Intervention: 62.2 (15.0) | Control: Male: 14 (63.6%)  Female: 8 (36.4%)    Intervention: Male 15 (62.5%)  Female: 9 (37.5%) |  | CES-D:  Control: 15.6 ± 9.9 Unlikely Depression  Intervention: 15.5 ± 13.2 Unlikely Depression |

**Psychotherapy**

| **First Author (Reference)** | **Mean Age (SD)** | **Gender** | **Ethnicity** | **Depression** |
| --- | --- | --- | --- | --- |
| **Picariello (32)** | 56.41 (17.86) | Female: 12 (50%) | Caucasian: 14 (58.3%)  Black: 6 (25.0%)  Asian: 4 (16.7%) | PHQ-9:  Control: 11.33 ± 4.52 Moderate Depression  Intervention: 13.41 Moderate Depression |
| **Shirazian(33)** | 59.9 (14.3) | Female: 17 (50%) | Non-Hispanic White: 9 (26.5%)  Non-Hispanic Black: 23 (67.6%)  American Indian/Alaska Native: 2 (6.3%) | PHQ-9: 5.3±4.5 Mild Depression |
| **Rahimipour(34)** | 47.82 (15.12) | Female (48%)  Male (52%) |  | PHQ-9:  Control: 13.64 ± 3.5 Moderate Depression  Intervention: 13.36 ± 3 Moderate Depression |
| **Bennett(35)** |  |  |  | Percentage of patients who reported depressive symptoms on PHQ-4:  Control: 22%  Intervention: 17% |
| **Nassim (36)** | 62.2 (12.0) | Female: 32 (58.2%) |  | PHQ-9:  Control: 14.7 ±5.2 Moderate Depression  Intervention: 12.8 ± 4.2 Moderate Depression |
| **Rigas(37)** | 61.1 (11.9) | Female: 37.5% |  | Percentage with depression based on PHQ-9:  Control: 9.5%  Intervention: 10.5% |
| **Thomas(38)** | 65 (13) | Female: 14 (33%) | White: 20 (49%)  Non-white: 21 (51%) | PHQ-9: 12.3±5.0 Moderate Depression |
| **Igarashi (44)** | Median Age: 69.5 (range, 31-88) | Male: 63.6% | Caucasian: 81.8%  Asian: 18.2% | BDI: Median with first and third quartiles: 7.00 [2.00; 11.00] Minimal Depression |
| **Shokrpour(39)** | Control: 62.82 (8.63)  Intervention: 58.97 (9.68) | Control:  Male: 60%  Female: 40%    Intervention:  Male: 77.1%  Female: 22.9% |  | DASS-21:  Control: 18.97±3.19 Moderate Depression  Intervention: 19.20±3.85 Moderate Depression |
| **González-Flores(40)** | 34 (12) | Female: 16 (31%)  Male: 36 (69%) |  | BDI:  Control: 17.48 ± 5.71 Mild Depression  Experimental: 18.88 ± 5.49 Mild Depression |
| **Nadort (41)** | 64 (15) | Male: 117 (62%) |  | BDI-II: 19.0 ± 7.7 Mild Depression |
| **Dingwall(42)** | 55 (9.4) | Male: 44 (28.2%)  Female: 112 (71.8%) |  | PHQ-9: 8.6 ± 5.2 Mild Depression |
| **Griva(43)** | 53.5 (10.4) | Female: 98 (41.7%) | Chinese: 133 (56.8%), Malay: 80 (34.2%), Indian: 15 (6.4%), Others: 6 (2.5%) | Baseline measured with HADS-D, but no precise quantitative value reported. |

**Music Therapy**

| **First Author (Reference)** | **Mean Age (SD)** | **Gender** | **Ethnicity** | **Depression** |
| --- | --- | --- | --- | --- |
| **Hagemann (48)** | 54.9 (14.6) | Female: 56.5%  Male: 43.5% | White: 69.5%  Black: 30.5% | BDI-II: 15.43 ± 9.2 Mild Depression |
| **Imani (45)** | Control: 56.21 ± 6.60  Intervention: 57.76 ± 6.74 | Control: Male (52%)  Intervention: Male (56%) |  | BDI:  Control: 23.2 ± 72.82 Moderate Depression  Intervention: 23.8 ± 68.99 Moderate Depression |
| **Bro (46)** | Control: 61 (14)  Intervention: 65 (20) | Control: Male: 75%  Intervention: Male: 50% |  | HADS-D  Control: 3.7 ± 2.4 Minimal Depression  Intervention: 2.9 ± 2.5 Minimal Depression |
| **Burrai(47)** | 62.3 (2.8) | Male: 62.5% |  | Baseline measured with HADS-D, but no precise quantitative value reported. |

**Abbreviations**: MADRS, Montgomery-Asberg Depression Rating Scale; CES-D, Center for Epidemiologic Studies Depression Scale; BDI-II, Beck’s Depression Inventory; ZSDS, Zung Self-Rating Depression Scale; PHQ-9, Patient Health Questionnaire-9; HADS-D, Hospital Anxiety and Depression Scale-Depression; PHQ-4, Patient Health Questionnaire-4; DASS-21, Depression, Anxiety, and Stress Scale-21
